# Advances of herbivore-secreted elicitors and effectors in plant-insect interactions

**DOI:** 10.3389/fpls.2023.1176048

**Published:** 2023-06-19

**Authors:** Huiying Wang, Shaojie Shi, Wei Hua

**Affiliations:** ^1^ Key Laboratory of Biology and Genetic Improvement of Oil Crops, Ministry of Agriculture and Rural Affairs, Oil Crops Research Institute of the Chinese Academy of Agricultural Sciences, Wuhan, China; ^2^ Hubei Hongshan Laboratory, Wuhan, China; ^3^ Hubei Key Laboratory of Food Crop Germplasm and Genetic Improvement, Key Laboratory of Crop Molecular Breeding, Ministry of Agriculture and Rural Affairs, Institute of Food Crops, Hubei Academy of Agricultural Sciences, Wuhan, China

**Keywords:** elicitors, effectors, defense responses, multi-omics approach, plant-insect interactions

## Abstract

Diverse molecular processes regulate the interactions between insect herbivores and their host plants. When plants are exposed to insects, elicitors induce plant defenses, and complex physiological and biochemical processes are triggered, such as the activation of the jasmonic acid (JA) and salicylic acid (SA) pathways, Ca^2+^ flux, reactive oxygen species (ROS) burst, mitogen-activated protein kinase (MAPK) activation, and other responses. For better adaptation, insects secrete a large number of effectors to interfere with plant defenses on multiple levels. In plants, resistance (R) proteins have evolved to recognize effectors and trigger stronger defense responses. However, only a few effectors recognized by R proteins have been identified until now. Multi-omics approaches for high-throughput elicitor/effector identification and functional characterization have been developed. In this review, we mainly highlight the recent advances in the identification of the elicitors and effectors secreted by insects and their target proteins in plants and discuss their underlying molecular mechanisms, which will provide new inspiration for controlling these insect pests.

## Introduction

Plants are constantly being attacked by various insects. Nearly half a million insect species live on plants (Wu and Baldwin, 2010). The vast majority of herbivorous insects feed on plants from a single taxonomic family or a few closely related plant species specifically, while only 10% of them establish intimacy with multiple plant species (Schoonhoven et al., 2005). In addition to the direct damage caused by feeding, insects can also injure plants indirectly by transmitting viral, bacterial, and fungal pathogens. The main strategy for crop protection against insects over the past several decades was the application of chemical insecticides. However, due to the emergence of insect resistance to pesticides and the negative effect on the environment, the use of such compounds has declined in recent years (Du et al., 2020). Scientists have begun to unravel the molecular mechanisms underpinning the interactions between plants and insects in order to find better ways to control these pests.

Over the years, evidence has been accumulated during the long-term interaction and evolution of plants and insects, and both host plants and insect herbivores have obtained diverse sophisticated mechanisms to adapt to each other. In general, the perception of insect attack is the first step of plant defenses. Insect elicitors are the biologically active molecules from insects’ saliva or gut regurgitant; they are recognized by plants and subsequently induce plant defenses (Chen and Mao, 2020; Snoeck et al., 2022). These elicitors are also called herbivore-associated molecular patterns (HAMPs) (Snoeck et al., 2022). The elicitor-induced defenses include depolarization of the plasma trans-membrane potential, activation of JA and SA pathways, ROS burst, callose deposition, Ca^2+^ influx, MAPK activation, etc. (Erb and Reymond, 2019; Ye et al., 2019; Li et al., 2019a). For successful infestation, insect herbivores secret salivary molecules into plant cells to weaken their defense responses; these active molecules are called effectors (Mutti et al., 2008; Bos et al., 2010; Hogenhout and Bos, 2011; Naessens et al., 2015; Rodriguez et al., 2017). Effectors that suppress the plant’s responses can be recognized by their corresponding resistance proteins, inducing a second layer of defense, the effector-triggered immunity (ETI) (Jones and Dangl, 2006; Takken and Tameling, 2009). Notably, the second layer of defense response is much more fierce than the first layer. In summary, the active molecules from insect secretion have a significant impact on plant immunity. The molecules that can trigger plant defense responses are defined as elicitors, while those that weaken plant defenses are called effectors (Chen and Mao, 2020). In this review, we mainly discuss the recent advances in research on elicitors and effectors secreted by insects and their roles in the interactions between insects and their host plants. Dissecting the plant host factors and pathways targeted by these active insect molecules will facilitate the characterization of the molecular mechanisms of plant-insect interactions.

## Herbivore feeding behaviors

To obtain nutrients from the hosts, insects employ diverse feeding strategies upon landing. Based on the different mouthparts and feeding habits, herbivorous insects can be divided into two groups: chewing and piercing-sucking insects (Schoonhoven et al., 1998; Walling, 2000). The insect species that cause damage with mouthparts evolved for chewing, snipping, or tearing belong to chewing insects, like leaf-eating beetles, caterpillars, or cotton bollworms. Chewing insects have a chewing type of mouth, which consists of the labrum, mandibles, first maxillae, second maxillae, hypopharynx, and epipharynx. The rectangular flap-like labrum is in the middle. The mandibles are paired and bear toothed edges at their inner surfaces; they masticate food using two sets of muscles transversely. The first maxillae and second maxillae are paired. The first maxillae are responsible for holding food and the second maxillae are responsible for pushing masticated food into the mouth. The hypopharynx has a single median tongue-like process, and the opening of the salivary duct lies under the hypopharynx. The epipharynx with taste buds is a single small membranous piece at the base of the labrum (Kahl, 1982; Felton et al., 1999; Stotz et al., 1999). Oral secretion (OS, consisting of regurgitant and saliva) of chewing insects contains active molecules that have a big impact on plant defense responses that are distinguishable from general mechanical damage (Hogenhout and Bos, 2011; Chen and Mao, 2020).

Piercing-sucking herbivorous insects, such as aphids, whiteflies, and planthoppers feed on plants through specially adapted mouthparts known as stylets, which they use to puncture the plant surface to access the phloem sap. The mouthparts of piercing-sucking insects are composed of the labrum, the labium, and the stylet. Among them, the stylet is used for piercing and sucking phloem sap from plants (Sogawa, 1982; Backus, 1988). The feeding strategies of piercing-sucking insects are mainly divided into three major phases, labial exploration, stylet penetration, and phloem-sap sucking (Spiller, 1990; Hao et al., 2008; Cheng et al., 2013b; Will et al., 2013). During their initial encounter with their host plants, insects walk rapidly and dab repeatedly on the plant’s surface to find a suitable feeding site, which is essential for the survival of the insects (Sogawa, 1982; Backus, 1988; Walling, 2008). Rice leaf sheath surface is featured in units and subunit structures, including the silico-phellem block, stomate block, large tubercle block, and vein, which are often covered with tubercle papicles, little papicles, glochids, and tenuous hairs. Recent research has shown that the brown planthopper (*Nilaparvata lugens* Stål, BPH), the most destructive pest of rice, preferentially selects the smooth long-cell block to probe their stylets into the leaf sheaths (Shi et al., 2021). Using an Atomic Force Microscope (AFM), Shi et al. (2021) found that the surface hardness of the long-cell block was much lower than that of the other cells. Sensilla basiconica, arranged symmetrically in two separate areas at the distal segment of the labium, was speculated to have a mechano-receptive function (Sogawa, 1982; Backus, 1988). Thus, we suppose that the labium may guide the stylets to find the suitable feeding site by sensing the mechanical heterogeneity of different structures on the plant surface.

Piercing-sucking insects penetrate plants with their stylet and move the stylet toward the phloem (Will et al., 2013). Along the stylet track, different types of cells are regularly penetrated (Will et al., 2013). Sucrose and pH are suggested to be indicators of phloem penetration (Hewer et al., 2010; Hewer et al., 2011). During the penetration process, piercing-sucking insects secrete both gelling and watery saliva from their salivary glands into the plant cells, and the protein compositions of the two types of saliva were shown to have some overlap (Walling, 2008; Huang et al., 2016). The secreted gelling saliva quickly solidifies and forms a continuous salivary sheath along its stylets for providing mechanical stability and protection (Wang et al., 2008). Some secretary proteins have been proven to be the key factors for forming the salivary sheath (Will and Vilcinskas, 2015; Huang et al., 2016; Huang et al., 2017; Shangguan et al., 2018; Huang et al., 2023). Watery saliva contains many active molecules that are involved in the induction or suppression of defenses against insect attack, i.e., the elicitors and effectors (Kaloshian and Walling, 2016; Chen and Mao, 2020).

## Multi-omics approach to identifying elicitors and effectors

Saliva is a complex mixture of biomolecules with potential roles in encounters with plant immune responses (Miles, 1999; Will et al., 2013). Functional approaches such as proteomics and transcriptomics have facilitated the high-throughput identification of elicitors/effectors in regurgitant or saliva from various insect species (Harmel et al., 2008; Bos et al., 2010; Cooper et al., 2011; Nicholson et al., 2012; Ji et al., 2013; Nicholson and Puterka, 2014; Huang et al., 2016; Liu X. et al., 2016; Huang et al., 2018; Rao et al., 2019; Huang et al., 2023). The majority of the reported elicitors or effectors discussed below were identified using multi-omics approaches. Here, we take the salivary proteome and transcriptome of *N. lugens* as examples. Through comparative transcriptome analysis of the salivary glands of TN1 and Mudgo populations, 352 genes were predicted to encode secretory proteins (Ji et al., 2013). Among them, endo-*β*-1,4-glucanase (NlEG1) and NlSEF1 play important roles in rice-BPH interactions (Ji et al., 2017; Ye et al., 2017). Huang et al. (2016) performed proteomic analyses combined with genomic and transcriptomic analysis and identified 202 secreted salivary proteins in *N. lugens*. RNA interference revealed that salivap-3 is required for forming the salivary sheath, while annexin-like5 and carbonic anhydrase are indispensable for BPH survival (Huang et al., 2016). Recently, 1140 protein-coding genes were predicted in the secretome of *N. lugens* by Rao et al. (2019). Sequence analysis and homology searches revealed the presence of both conserved and rapidly evolving salivary proteins. Furthermore, six *N. lugens* secreted elicitors (Nl12, Nl16, Nl28, Nl32, Nl40, and Nl43) were identified by a series of predictions and functional analysis, as discussed below (Rao et al., 2019). The high-throughput identification of these secreted salivary proteins provides the possibility of understanding some aspects of plant-insect molecular interaction mechanisms and identifying potential targets for pest management.

## Insect-associated elicitors

In general, plants can recognize elicitors and produce a complex series of defenses. The first reported elicitor *β*-glucosidase was isolated from the regurgitant of the white butterfly (*Pieri brassicae*). Leaves treated with *β*-glucosidase enhanced the emission of volatiles that are highly attractive to the parasitic wasp (Mattiacci et al., 1995). The glucose oxidase (GOX) present in the saliva extracted from Noctuid caterpillars (*Helicoverpa zea*) and European corn borer (*Ostrinia nubilalis*) upregulates the expression of genes related to the JA biosynthesis pathway and the late responding defense, such as *proteinase inhibitor 2* (*Pin2*) in tomato (Tian et al., 2012; Louis et al., 2013). External spraying of phospholipase C (PLC), a salivary protein from fall armyworm (*Spodoptera frugiperda*), activates defense responses in maize and Bermuda grass and reduces caterpillar weight gain (Acevedo et al., 2018).

Except for the elicitors isolated from chewing insects, some elicitors were identified in piercing-sucking insects. Mp10 and Mp42 were two elicitors that were identified using a functional genomics approach in aphids. Aphid fecundity decreased when feeding on plants over-expressing *Mp10* and *Mp42*. In addition, Mp10 specifically induced chlorosis in *N. benthamiana* leaves in a SUPPRESSOR OF G2 ALLELE OF *skp1* (SGT1)-dependent manner (Bos et al., 2010; Rodriguez et al., 2014). Cysteine protease Cathepsin B3 (CathB3) was also recognized as a potential elicitor protein, which suppresses aphid feeding by triggering ROS through interacting with an ENHANCED DISEASE RESISTANCE 1-like (EDR1-like) protein (Guo et al., 2020). The mucin-like salivary protein (NlMLP) is a dual-functional protein both for insects and plants. In BPH, NlMLP is required for the formation of salivary sheath. In plants, NlMLP induces cell death, the expression of defense-related genes, and callose deposition (Shangguan et al., 2018). When BPH feed or oviposit, the small N-terminal subunit of vitellogenins (VgN) induces strong defenses, such as ROS burst and other responses in rice (Zeng et al., 2023). The ectopic expression of six secreted salivary proteins from BPH (Nl12, Nl16, Nl28, Nl32, Nl40, and Nl43) could induce cell death, chlorosis, or a dwarf phenotype, respectively in *N. benthamiana* leaves (Rao et al., 2019). Some salivary proteins from other piercing-sucking insects were also identified as the elicitors, like Tet1, Tet2, disulfide isomerase (TetPDI) from spider mite (*Tetranychus evansi*) (Iida et al., 2019; Cui et al., 2023), and RP309 from Fabricius (*Riptortus pedestris*) (Dong et al., 2022). It is noteworthy that although elicitor-induced plant defenses impair the performance of insects on plants, RNA interference (RNAi) experiments have revealed that elicitors are still essential for the survival of insects (Shangguan et al., 2018; Guo et al., 2020; Cui et al., 2023; Zeng et al., 2023).

In addition to the elicitors coming from insects themselves, some elicitors are generated from the microbes they carry. *Buchnera aphidicola* is the endosymbiont of potato aphids (*Macrosiphum euphorbiae*). Over-expression of *Buchnera GroEL* in Arabidopsis plants induces ROS burst and PTI, which is associated with the BRASSINOSTEROID INSENSITIVE1-ASSOCIATED RECEPTOR KINASE 1 (BAK1), thus reducing the fecundity of the aphid (Chaudhary et al., 2014). A porin-like protein (PLP) from bacteria in oral secretions of *Spodoptera littoralis* larvae induces Ca^2+^ flux *in vitro* and upregulates the calmodulin-like CML42 (Guo et al., 2013).

**Figure 1:**
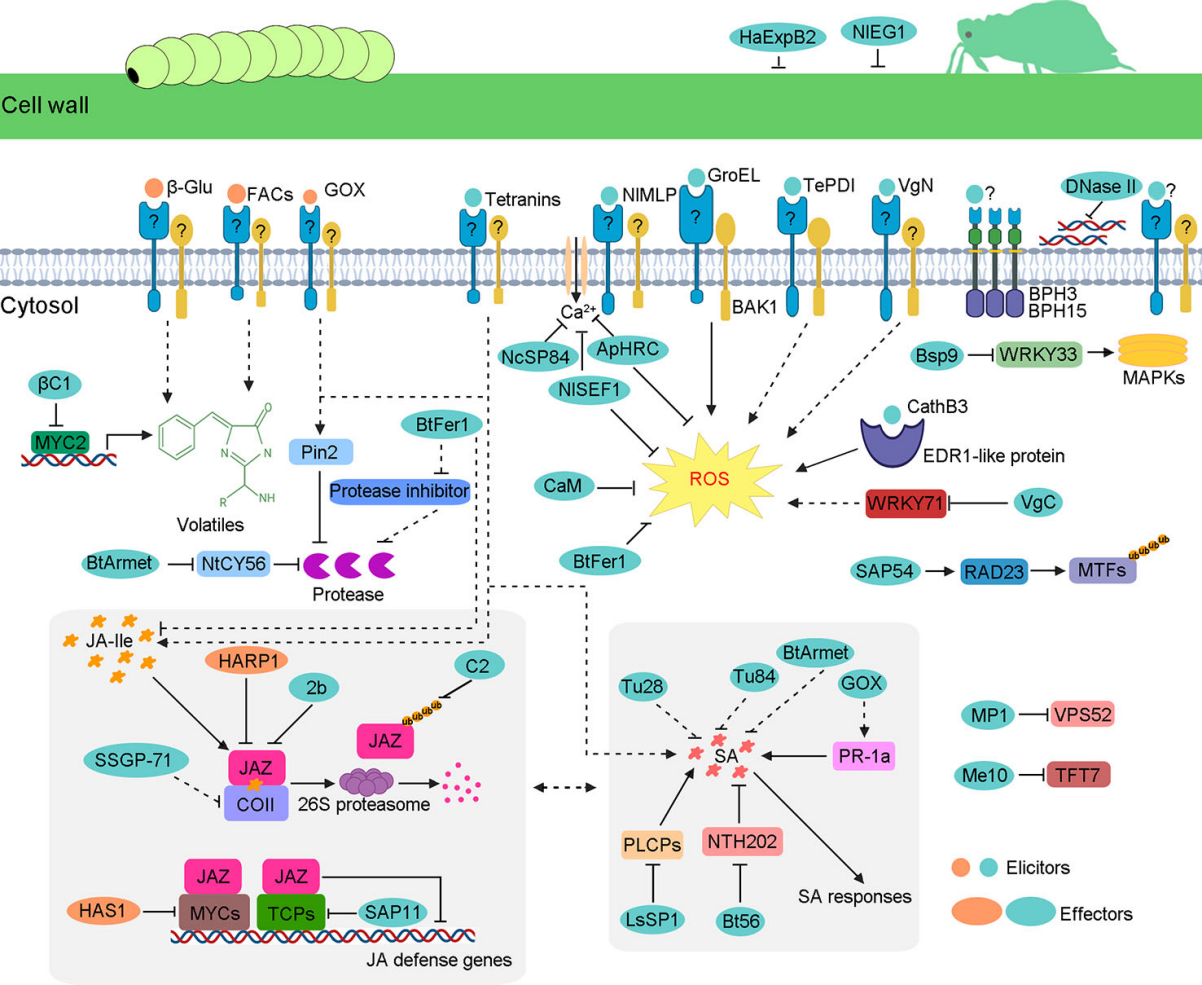
Schematic model of insect-secreted elicitors and effectors regulating plant defenses. When insects feed on plants, elicitors induce a complex series of plant defenses, such as ROS burst; upregulation of JA, SA and some volatile; and other unknown responses. However, insects secrete effectors to suppress these defense responses. Some effectors weaken JA pathways, including HARP1, HAS1, 2b, C2, *β*C1, SAP11, SSGP-71, and BtFer1. Some effectors interfere with SA pathways, such as BtArmet, Bt56, GOX, and LsSP1. The same effector protein can participate in diverse defense pathways. For example, as well as the reduction of the Ca^2+^ influx, ApHRC and NlSEF1 also suppress ROS burst. The *B. tabaci* effector BtFer1 not only reduces the accumulation of ROS and JA but also weakens protease inhibitor activity, thus increasing the content of protease to help whitefly feed better. Similar to BtFer1, effector BtArmet interacts with protease inhibitor NtCY56 to block the inhibition of whitefly protease. Mp1 and Me10 target plant proteins VPS52 and TFT7, respectively, which are required for insect resistance. The DNase II targets the extracellular DNA that is released by damaged cells. NlEG1 and HaExPB2 enable the insect’s stylet to reach the phloem by degrading celluloses in host plant cell walls.

Some elicitors are relatively conserved in their ability to induce responses across a range of plant species. Both Nl32 in planthopper and MP10 in aphids are chemosensory proteins (CSPs), small water-soluble proteins with an OS-D domain that are conserved among different insects (Pelosi et al., 2005; Bos et al., 2010; Rao et al., 2019). Eleven CSPs (NlCSP-1 to -11) were previously identified in BPH, and six out of the eleven CSPs induced similar effects on *N. benthamiana* to those caused by Nl32 and Mp10 (Bos et al., 2010; Zhou et al., 2015; Rao et al., 2019). Nl12 and TetPDI, deriving from planthopper and spider mite, respectively, are the two members of the conserved disulfide isomerase family in eukaryotic organisms (Rao et al., 2019; Cui et al., 2023). Moreover, PDIs from phylogenetically distinct herbivorous and non-herbivorous arthropods could induce plant immunity in an SGT1/HSP90-dependent way (Cui et al., 2023). GOX was also conserved among caterpillar species (Tian et al., 2012; Louis et al., 2013). In **Figure 1** and **Table 1**, we summarize the reported insect-associated elicitors from different species and their respective roles.

**Table 1:** Insect-associated elicitors.

Name	Origin	Protein characterization	Function	Reference
*β*-glu	*Pieri brassicae*	*β*-glucosidase	Release attractive volatiles to parasitic wasps (*Cotesia glomerata*)	Mattiacci et al., 1995
Caeliferins	*Schistocerca* *americana*	Disulfooxy fatty acids	Induce volatile emissions in corn	Alborn et al., 2007
GOX	*Helicoverpa zea*; European corn borer	Glucose oxidase	Elicit JA pathway and late responding defenses in tomato	Tian et al., 2012; Louis et al., 2013
PLC	*Spodoptera frugiperda*	Phospholipase C	Reduce caterpillar weight gain; Induce defense responses in maize and Bermuda grass	Acevedo et al., 2018
Mp10	*Myzus persicae*	Chemosensory protein	Reduce aphid fecundity in tobacco	Bos et al., 2010
CathB3	*Myzus persicae*	Cysteine protease	Reduce aphid performance; Induce ROS burst in an EDR1-dependent manner in tobacco	Guo et al., 2020
NlMLP	*Nilaparvata lugens*	Mucin-like protein	Salivary sheath formation; Induce plant defense response in rice and tobacco	Shangguan et al., 2018
Nl12	*Nilaparvata lugens*	Disulfide isomerase	Induce cell death, expression of defense-related genes, and callose deposition in tobacco	Rao et al., 2019
Nl16	*Nilaparvata lugens*	Apolipophorin-III protein	Induce cell death, expression of defense-related genes, and callose deposition in tobacco	Rao et al., 2019
Nl28	*Nilaparvata lugens*	Cysteine-rich protein	Induce cell death, expression of defense-related genes, and callose deposition in *Nicotiana benthamiana*	Rao et al., 2019
Nl32	*Nilaparvata lugens*	Chemosensory protein	Induce a dwarf phenotype, expression of defense-related genes, and callose deposition in tobacco	Rao et al., 2019
Nl40	*Nilaparvata lugens*	*N.lugens-*specific salivary protein	Induce chlorosis, expression of defense-related genes, and callose deposition in tobacco	Rao et al., 2019
Nl43	*Nilaparvata lugens*	Uncharacterized protein	Induce cell death, expression of defense-related genes, and callose deposition in tobacco	Rao et al., 2019
VgN	*Nilaparvata lugens*	N-terminal subunit of vitellogenin	Trigger strong defense responses in rice	Zeng et al., 2023
Te1	*Tetranychus evansi*	Tetranins	Induce JA, SA, and ABA biosynthesis in tobacco	Iida et al., 2019
Te2	*Tetranychus evansi*	Tetranins	Induce JA, SA, and ABA biosynthesis in tobacco	Iida et al., 2019
TePDI	*Tetranychus evansi*	Disulfide isomerase	Reduce aphid performance; Induce ROS burst, callose deposition, expression of defense-related genes, and cell death in an SGT1/HSP90-dependent manner in tobacco	Cui et al., 2023
RP309	*Riptortus pedestris*	*R. pedestris*-specific salivary protein	Induce cell death, ROS burst, and the expression of PTI marker genes in tobacco	Dong et al., 2022
PLP	Bacteria *in Spodoptera littoralis*	Porin-like protein	Induce defense-related early events in *Arabidopsis*	Guo et al., 2013
GroEL	*Buchnera aphidicola* in *Macrosiphum* *euphorbiae*	Chaperonin	Reduce aphid fecundity; Induce ROS burst and expression of PTI marker genes in *Arabidopsis*	Chaudhary et al., 2014

## Effectors involved in plant-insect interactions

To adapt to their host plants, insects secrete a repertoire of effectors to disturb host plant defense responses (**Figure 1** and **Table 2**). GOX from caterpillar *H. zea* was the first reported insect effector. The nicotine accumulation was suppressed significantly by GOX in tobacco (Musser et al., 2002; Musser et al., 2005). Interestingly, the same GOX was characterized as an elicitor in the ‘Insect-associated elicitors’ section because it induces plant responses in tomato (Tian et al., 2012; Louis et al., 2013). These results indicate that the same protein can act as the effector or as the elicitor when encountering different host plants. The cotton bollworm (*Helicoverpa armigera*) is a destructive lepidopteran insect widely existing in agriculture. Chen et al. (2019) identified an effector, a venom-like protein termed HARP1, from the OS of *H. armigera*. HARP1 stabilizes JAZ degradation and blocks wound-induced JA signaling transduction by forming a protein complex with JAZ. The weight of *H. armigera* larvae was increased significantly on transgenic plants with high-level *HARP1* (Chen et al., 2019). HAS1 is another effector of *H. armigera*. Plants over-expressing *HAS1* exhibit more susceptibility to insect herbivores accompanied by the suppressed JA pathway due to the interactions between HAS1 and JASMONATE-ZIM-domain repressors MYC3/MYC4 (Chen et al., 2023). These results indicate that interfering with the JA pathway is a common strategy of effectors in chewing insects.

**Table 2:** Identified insect-associated effector proteins.

Name	Origin	Protein characterization	Function	Reference
GOX	*Helicoverpa zea*	Glucose oxidase	Inhibit the production of nicotine in tobacco	Musser et al., 2002
HARP1	Helicoverpa armigera	Venom R-like protein	Enhance cotton bollworm feeding performance; Block JA pathway by interacting with JAZ in *Arabidopsis*	Chen et al., 2019
HAS1	*Helicoverpa armigera*	Venom R-like protein	Enhance cotton bollworm feeding performance; Block JA pathway by interacting with MYC3/MYC4 in *Arabidopsis*	Chen et al., 2023
C002	*Myzus persicae*	Salivary glands-abundant secretory protein	Promote aphid colonization in tobacco	Pitino and Hogenhout, 2013
Mp55	*Myzus persicae*	Salivary glands-abundant secretory protein	Increase aphid reproduction; Reduce accumulation of 4-methoxyindol-3-ylmethylglucosinolate, callose, and hydrogen peroxide in tobacco	Elzinga et al., 2014
Mp1	*Myzus persicae*	Salivary glands-abundant secretory protein	Increase aphid reproduction; Target trafficking protein VPS52 in tobacco	Rodriguez et al., 2017
MIF	*Acyrthosiphon* *Pisum*; *Myzus persicae*	Macrophage migration inhibitory factor	Enable aphid survival, fecundity, and feeding; Suppress *Cry*-triggered defenses in tobacco	Naessens et al., 2015
Me10	*Macrosiphum euphorbiae*	Salivary glands-abundant secretory protein	Enhance aphid fecundity; Suppress defenses and interact with tomato TFT7 in tomato	Atamian et al., 2013; Chaudhary et al., 2019
Me23	*Macrosiphum euphorbiae*	Glutathione peroxidase	Suppress plant defenses in tobacco	Atamian et al., 2013
ACE1 and ACE2	*Acyrthosiphon pisum*	Angiotensin-converting enzymes	Enable aphid feeding and survival in tobacco	Wang et al., 2015b
Armet	*Acyrthosiphon pisum*	Arginine-rich, mutated in early stage of tumors	Enable aphid feeding; Elicit SA pathway in tobacco	Wang et al., 2015a; Cui et al., 2019
ApHRC	*Acyrthosiphon pisum*	Histidine-rich Ca^2+^- binding like protein	Promote aphid colonization; Repress Ca^2+^ elevation and ROS accumulation	Wang et al., 2020
Sg2204	*Schizaphis graminum*	Salivary glands-abundant secretory protein	Enable aphid feeding; Suppress JA, SA pathways, and cell death caused by BAX/INF1 in tobacco	Zhang et al., 2022a
Sm9723	*Sitobion miscanthi*	Salivary glands-abundant secretory protein	Enable aphid feeding; Suppress JA, SA pathways, and BAX/INF1-induced cell death in tobacco	Zhang et al., 2022b
NlEG1	*Nilaparvata lugens*	Endo-*β*-1,4-Glucanase	Enable BPH feeding; Degrade celluloses in rice	Ji et al., 2017
NlSEF1	*Nilaparvata lugens*	EF-hand calcium-binding protein	Suppress the production of Ca^2+^ and H_2_O_2_ in rice	Ye et al., 2017
NlugOBP11	*Nilaparvata lugens*	Odorant-binding protein	Enable BPH feeding; Suppress SA pathway in rice	Liu et al., 2021
CaM	*Nilaparvata lugens*; *Laodelphax striatellus*	Calmodulin binding protein	Enable BPH fecundity; Suppress H_2_O_2_ accumulation, and callose deposition in rice	Fu et al., 2022
NlSP7	*Nilaparvata lugens*	Salivary glands-abundant secretory protein	Enable BPH feeding; Mediate tricin metabolism in rice	Gong et al., 2022
DNase II	*Laodelphax striatellus*	DNase II	Enable SBPH feeding performance; Reduce H_2_O_2_ and callose accumulation in rice	Huang et al., 2019
VgC	*Laodelphax striatellus*	C-terminal peptide of vitellogenin	Suppress H_2_O_2_ accumulation by targeting OsWRKY71 in rice	Ji et al., 2021
LsSP1	*Laodelphax striatellus*	Salivary glands-specific protein	Enable SBPH feeding performance; Reduce SA responses by interacting with PLCPs in rice	Huang et al., 2023
LAC1	*Bemisia tabaci*	Laccase	Enable whitefly survival; Upregulated by JA signaling in tomato	Yang et al., 2017
BtFer1	*Bemisia tabaci*	Ferritin	Enable whitefly survival; Suppress JA pathway in tomato	Su et al., 2019
Bsp9	*Bemisia tabaci*	Salivary glands-abundant secretory protein	Promote whitefly performance; Suppress plant defenses by interacting with WRKY33 tobacco	Wang et al., 2019
Bt56	*Bemisia tabaci*	Low molecular weight salivary protein	Promote whitefly performance; Elicit SA pathway by targeting tobacco NTH202	Xu et al., 2019
BtArmet	*Bemisia tabaci*	Arginine-rich, mutated in early stage of tumors	Enhance whitefly performance; Target tobacco NtCYS6	Du et al., 2022
Tu28	*Tetranychus urticae*	Protein with Armadillo- type fold domain	Promote spider mite performance; Suppress SA-pathway in tobacco	Villarroel et al., 2016
Tu84/Te84	*Tetranychus urticae*; *Tetranychus evansi*	Salivary glands-abundant secretory protein	Promote spider mite performance; Suppress SA pathway in tobacco	Villarroel et al., 2016
NcSP84	*Nephotettix* *cincticeps*	EF-hand calcium-binding protein	Bind Ca^2+^ ions and facilitate stylet puncturing in rice	Hattori et al., 2012
NcSP75	*Nephotettix* *cincticeps*	Salivary glands-specific protein	Enable leafhopper survival and feeding performance in rice	Matsumoto and Hattori, 2018
vH13	*Mayetiola* *destructor*	*M. destructor-*specific salivary protein	Elicit effector-triggered immunity in resistant wheat containing *H13*	Aggarwal et al., 2014
SSGP-71	*Mayetiola* *destructor*	E3-ubiquitin-ligase mimic	Target Skp in wheat	Zhao et al., 2015
vH6	*Mayetiola* *destructor*	E3-ubiquitin-ligase mimic with an F box and 13 LRRs	Elicit effector-triggered immunity in resistant wheat containing *H6*	Zhao et al., 2015
vH9	*Mayetiola* *destructor*	E3-ubiquitin-ligase mimic without F box	Elicit effector-triggered immunity in resistant wheat containing *H9*	Zhao et al., 2015
SAP11	Aster Yellows phytoplasma *in Macrosteles quadrilineatus*	A 9-kDa protein	Promote leafhopper performance; Bind and destabilize TCP to suppress JA synthesis in *Arabidopsis*	Sugio et al., 2011
SAP54	Aster Yellows phytoplasma *in Macrosteles*	A 10.7-kDa protein	Promote leafhopper colonization; Degrade MTFs by interacting with RAD23 in *Arabidopsis*	MacLean et al., 2011; MacLean et al., 2014
2b	Cucumber mosaic virus in *Myzus persicae*	Virus protein	Interact with JAZ1 to suppress JA signaling in tobacco	Wu et al., 2017
*β*C1	Tomato yellow leaf curl China virus in *Bemisia tabaci*	Virus protein	Promote whitefly performance; Repress terpenoid synthesis by binding to MYC2	Luan et al., 2013; Li et al., 2014
C2	Tomato yellow leaf curl virus in *Bemisia tabaci*	Virus protein	Promote whitefly survival and reproduction; Suppress plant defenses by interacting with plant ubiquitin	Li et al., 2019b

Like those in chewing insects, effectors in piercing-sucking insects also disturb plant hormone-related defense pathways. Bt56 from the whitefly (*Bemisia tabaci*) increases susceptibility to insects by enhancing the accumulation of SA but not JA. Interaction assays have shown that Bt56 interacts directly with a KNOTTED 1-like homeobox transcription factor NTH202 (Xu et al., 2019). The survival rate and fecundity were significantly lower in insects injected with ds*Bt56* than in those injected with ds*GFP* (Xu et al., 2019). BtArmet, another effector of the *B. tabaci*, increased whitefly performance on tobacco plants by suppressing SA accumulation and binding to the cystatin NtCYS6, a protease inhibitor that prevents insects from continuous ingestion and digestion (Du et al., 2022). BtFer1 is a *B. tabaci* salivary protein with Fe^2+^ binding ability. The results showed that BtFer1 suppressed the JA-mediated signaling pathway, ROS burst, callose deposition, and accumulation of proteinase inhibitors (Su et al., 2019). The small brown planthopper (*Laodelphax striatellus*, SBPH) effector LsSP1 not only binds to sheath protein LsMLP to avoid LsMLP protein being recognized by plants but also interacts with rice papain-like cysteine proteases to inhibit SA biosynthesis and SA-related defenses (Huang et al., 2023).

Moreover, some effectors were reported to target other defense-related pathways in plants. The *L. striatellus* secretes effector protein DNase II to inhibit defense responses by erasing extracellular DNA and reducing hydrogen peroxide (Huang et al., 2019). Interestingly, unlike the VgN, as a reliable elicitor, the C-terminal peptide of vitellogenin (VgC) acts as a novel effector in *L. striatellus*, which attenuates H_2_O_2_-mediated plant defense by interacting directly with the host transcription factor OsWRKY71 for promoting insect performance (Ji et al., 2021; Zeng et al., 2023). Salivary protein 7 (NlSP7), a salivary protein secreted from the brown planthopper, functions as an effector via mediating tricin metabolism in rice plants (Gong et al., 2022). TFT7, 14-3-3 isoform 7, has been proven to be required for aphid resistance in tomato. *Macrosiphum euphorbiae* saliva-secreted protein Me10 targets the TFT7 as an effective infestation strategy (Chaudhary et al., 2019). Interaction assays have shown that the effector Mp1 from *M. persicae* associates with the host Vacuolar Protein Sorting Associated Protein52 (VPS52), which has a negative impact on insect infestation (Rodriguez et al., 2017). Effector Bsp9 from *B. tabaci* interacts with WRKY33 to interfere with the association between WRKY33 and a central regulator in the MAPK cascade, thus inhibiting plant immunity (Wang et al., 2019). The SSGP-71 (Secreted Salivary Gland Proteins-71) family, which has 426 members, has the greatest representation in the salivary proteome of the Hessian fly. Most SSGP-71 genes encode proteins with a signal peptide and an F box domain, which interacts with an Skp1-like protein (Zhao et al., 2015). The host plant cell wall was the first barrier of defense against herbivores (Calderón-Cortés et al., 2012). Both nematode (*Heterodera avenae*) expansin-like protein (HaEXPB2) and brown planthopper NlEG1 target the cell wall for promoting insect performance (Liu J. et al., 2016; Ji et al., 2017). Some effectors can suppress elicitor/pathogen-associated molecular pattern (PAMP)-triggered immunity. A macrophage migration inhibitory factor (MIF) is secreted from aphid saliva to promote insect feeding. Further study revealed that over-expressing *MIF* inhibits defense responses caused by the elicitor cryptogein, a 10-kDa protein from the plant pathogen *Phytophthora cryptogea* (Naessens et al., 2015). Transient overexpression of the salivary effector SG2204 from greenbug (*Schizaphis graminum*) and Sm9723 from grain aphid (*Sitobion miscanthi*) could suppress BAX and PAMP INF1-induced cell death (Zhang et al., 2022a; Zhang et al., 2022b). Furthermore, spider mite effectors Te28 and Te84 could also suppress cell death caused by the elicitor TePDI (Cui et al., 2023). However, as yet, the targets or receptors of many effectors in plants have not been identified.

Like elicitors, some effectors also come from insect-borne microbes. Notable examples are the SAP11 and SAP54 from Aster Yellows phytoplasma strain Witches’ Broom (AY-WB). They alter plant development and defense responses by the destabilization of CINCINNATA (CIN)-related TEOSINT BRANCHED1/CYCLOIDEA/PROLIFERATING CELL FACTOR (TCP) and MADS domain transcription factors (MTFs) to enhance insect vector reproduction (MacLean et al., 2011; Sugio et al., 2011; MacLean et al., 2014). Other microbe-derived effectors, such as C2 from tomato yellow leaf curl China virus and 2b from cucumber mosaic virus (CMV), promote insect vector infestation by blocking the JA pathway in the plant (Wu et al., 2017; Li et al., 2019b). Together, these examples illustrate that microbe-derived effectors contribute to facilitating the fitness of their insect vectors as an effective strategy for completing their infection cycles.

## R gene-mediated plant resistance to insect herbivores

To fight the secreted effectors, host plants have developed resistance proteins. A set of genes in tomato, melon, and rice conferring resistance against insects has been identified and cloned. Two aphid resistance genes, the *Mi-1.2* gene characterized in the tomato (*Solanum lycopersicum*) and the *Vat* gene characterized in the melon (*Cucumis melo*) confer resistance to the potato aphid (*M. euphorbiae*) and the cotton aphid (*A.gossypii*), respectively (Rossi et al., 1998; Vos et al., 1998; Dogimont et al., 2014). Besides the potato aphid, the *Mi-1.2* gene is also resistant to two whitefly biotypes, a psyllid, and three nematode species, suggesting that the *Mi-1.2* gene confers a broad-spectrum resistance (Vos et al., 1998). With the availability of genome sequence data and molecular markers in rice, research on BPH-resistance genes has made a spurt of progress. BPH-rice interaction has become an excellent model system for the study of plant-insect interactions and co-evolution (Jing et al., 2017). To date, a total of 17 genes conferring resistance to BPH (*Bph1*, *Bph2*, *Bph3*, *Bph6*, *Bph7*, *Bph9*, *Bph10*, *Bph14*, *Bph15*, *Bph18*, *Bph21*, *Bph26*, *bph29*, *Bph30*, *Bph32*, *Bph37*, *Bph40*) have been cloned and characterized in rice plants (Du et al., 2020; Muduli et al., 2021; Shi et al., 2021; Zhou et al., 2021), which has shed a light on the molecular basis of plant-insect interactions.

*Bph14*, which encodes a typical NLR protein, was the first isolated BPH-resistance gene (Du et al., 2009). Further research has revealed that BPH14 protein stabilizes WRKY46 and WRKY72 to increase the expression of the receptor-like cytoplasmic kinase gene *RLCK281* in rice (Hu et al., 2017). *Bph9*, a BPH-resistance gene mapped on the long arm of rice chromosome 12 (12L), which is allelic with another seven BPH-resistance genes (*Bph1*, *Bph2*, *Bph7*, *Bph10*, *Bph18*, *Bph21*, and *Bph26*), encodes an unusual NLR protein that confers resistance to BPH by enhancing SA and JA signaling pathways (Zhao et al., 2016). BPH6, an uncharacterized protein that localizes to the exocyst, interacts with the exocyst subunits OsEXO70E1 and OsEXO70H3, increases exocytosis, and participates in cell wall maintenance and reinforcement (Guo et al., 2018; Wu et al., 2022). Recently, a novel dominant BPH-resistance gene, *Bph30*, was isolated from the short arm of rice chromosome 4 (4S) (Wang et al., 2018; Shi et al., 2021). *Bph30* is strongly expressed in sclerenchyma cells and encodes a protein belonging to a novel gene family with two leucine-rich domains (LRDs). A functional study showed that BPH30 enhances cellulose and hemicellulose synthesis, making the cell walls stiffer and sclerenchyma thicker to prevent stylets from penetrating the leaf sheath tissue, thereby conferring broad resistance to BPH and WBPH in rice (Shi et al., 2021). *Bph15* encodes a lectin receptor-like kinase (LecRK), which functions in both innate immunity and seed germination in plants (Cheng et al., 2013a). *Bph3* consists of a cluster of three genes encoding the plasma membrane-localized LecRKs (OsLecRK1, OsLecRK2, and OsLecRK3), which have a cumulative effect on resistance (Liu et al., 2015). These results indicate the diversity in resistance genes and mechanisms.

Based on our knowledge, except for two lectin receptor-like receptors and a few R proteins with unusual structures, such as BPH6 and BPH30, most isolated BPH-resistance proteins belong to nucleotide-binding and leucine-rich repeat (NLR) proteins, suggesting commonality between the perception of phloem-feeding insects and pathogens by plants. *Bph3* and *Bph15* encode the LecRKs, which resemble pattern recognition receptors (PRRs). PRRs are activated in response to microbe/pathogen/herbivore-associated molecular patterns or apoplastic effectors (Kaloshian and Walling, 2016; Ngou et al., 2022). The first layer of resistance to BPH may be BPH3 or BPH15, which is activated by the recognition of elicitors or apoplastic effectors. The second layer of resistance to BPH may be BPH6, BPH14, and BPH9 and their alleles, which can specifically recognize their cognate effectors and trigger defense responses (Jing et al., 2017; Du et al., 2020; Zheng et al., 2021).

## Effectors recognized in R protein-mediated resistance

Despite the recent insights into the complex repertoire of R proteins, only a few effectors recognized by R proteins have been identified until now. This may be owing to the genetic intractability of the insects. At least 40 brown planthopper-resistant genes have been discovered, but just four corresponding BPH effector loci (*Qhp7*, *Qgr5*, *Qgr14*, and *vBph1*) were mapped (Jing et al., 2014; Kobayashi et al., 2014). The effectors recognized by R proteins had only been isolated from Hessian fly (*Mayetiola destructor*) until now (Stuart, 2015). The first Hessian fly virulence gene, *virulence to Hessian fly 13* (*vH13*), was isolated using a map-based cloning strategy (Rider et al., 2002; Aggarwal et al., 2009; Aggarwal et al., 2014). Functional assays have shown that *vH13* transcripts are only detected in *H13*-avirulent larvae and are lost in *H13*-virulent larvae. RNAi results revealed that the knockdown of *vH13* helped some *H13*-avirulent larvae to escape the resistance triggered by *H13* in wheat. Furthermore, *vH13* encodes a small modular protein with no sequence similarities to other proteins in the database (Aggarwal et al., 2014).

Two additional Hessian fly effectors, vH6 and vH9, were identified by the completion of the Hessian fly genome sequencing and gene expression analyses. vH6 and vH9 can overcome the resistance mediated by wheat R protein H6 and H9, respectively (Zhao et al., 2015). Both *vH6* and *vH9* encode SSGP-71-like proteins. In H6-virulent Hessian flies, an SSGP-71 gene (*Mdes009086-RA*) is lost, suggesting Mdes009086-RA is the cognate effector of H6. In H9-virulent Hessian flies, two candidate SSGP-71 proteins without F-box domains were perfectly associated with H9 virulence, especially candidate 2 (Mdes015365-RA). These results indicate that the SSGP-71 family may play an essential role in the evolution of Hessian fly biotypes (Zhao et al., 2015). However, no cognate Hessian fly R protein has been cloned successfully, and the recognition mechanism of these Hessian fly effectors by the cognate R protein remains to be explored.

## Perspectives and challenges

In recent years, rapid technological progress in the discovery and interrogation of plant and insect genomes, transcriptomes, and proteomes has been made. These developments have provided opportunities for the exploration of molecules delivered by herbivores that activate or suppress plant immunity (Hogenhout and Bos, 2011; Kaloshian and Walling, 2016). Cas9-CRISPR and RNAi technologies can effectively silence host plant/insect genes and, therefore, can help us to reveal the important signal molecules and the key pathways in plant-insect interactions (Ma et al., 2015; Liu et al., 2020; Hough et al., 2022). These discoveries give us an advanced understanding of the plant-insect relationship. In particular, the RNAi tool has made important contributions to the study of the function of insect elicitors and effectors in insect performance and plant immunity. There are several strategies for the delivery of double-stranded RNA (dsRNA), including external spraying, artificial feeding/micro-injection of synthesized dsRNA, and construction of transgenic plant lines with high levels of endogenous dsRNA (Shangguan et al., 2018; Huang et al., 2020; Zhang et al., 2022a). Over the years, RNAi has been considered an effective strategy for the control of insect pests (Liu et al., 2020; Hough et al., 2022). In addition, great progress has been made in the research of insect resistance proteins, especially the resistance mechanism of BPH-resistant genes (Jing et al., 2017; Du et al., 2020; Zheng et al., 2021).

Despite these advances, major gaps in our understanding of interactions between insect herbivores and host plants remain to be filled. Although a large number of elicitors and effectors have been identified, only a few have revealed corresponding host targets. The plant defense pathways interfered with by the majority of elicitors and effectors are obscure. Additionally, little is known about the relationship between cognate insect effector and cognate R protein. On the one hand, no cognate effector of the cloned R genes has been discovered; on the other hand, while three R protein-recognized effectors in Hessian flies were identified, no corresponding Hessian fly R genes have been cloned. Combining map-based cloning and multi-omics approaches may contribute to overcoming these challenges. We believe that these questions will be the priority of research on plant-insect interactions in the next decade, and the answers to these questions will provide more insight into how to control these pests.

## Author contributions

WH proposed the idea. HW and SS drafted the manuscript and designed the figure. WH reviewed and edited the manuscript. All authors contributed to the article and approved the submitted version.
